# Forearm Magnetic Resonance Imaging Findings in Epidemic Myalgia

**DOI:** 10.31662/jmaj.2024-0425

**Published:** 2025-03-21

**Authors:** Remon Sato, Naohiro Uchio, Hirotaka Takagi, Hideyuki Matsumoto

**Affiliations:** 1 Department of Neurology, Mitsui Memorial Hospital, Tokyo, Japan; 2 Research Center for Biosafety, Laboratory Animal and Pathogen Bank, National Institute of Infectious Diseases, Tokyo, Japan

**Keywords:** epidemic myalgia, parechovirus A3, STIR hyperintensity

A 34-year-old man presented with myalgia after a fever. Neurological examination revealed myalgia in the posterior upper arms, radial forearms, and posterior lower extremities (Figure 1) and muscle weakness in the distal upper extremities, including the wrist flexor, wrist extensor, flexor digitorum superficialis, and flexor digitorum profundus muscles. Laboratory data showed elevated creatine kinase (1927 IU/L). Magnetic resonance imaging (MRI) indicated short-tau inversion recovery (STIR) hyperintensity in the radial forearm muscles and fascia (Figure 2). Epidemic myalgia was diagnosed by detecting parechovirus A3 ribonucleic acid in the patient’s stool. Epidemic myalgia typically shows limb myalgia and decreased grip strength ^(1)^. Although STIR hyperintensity in the muscles and fascia in the posterior upper arm and thigh is documented, forearm MRI findings have not been reported ^(1), (2), (3), (4)^. The radial forearm STIR hyperintensity aligns with myalgia site rather than muscle weakness. Further studies incorporating forearm findings are needed to clarify the relationship between MRI findings and myalgia.

**Figure 1.**
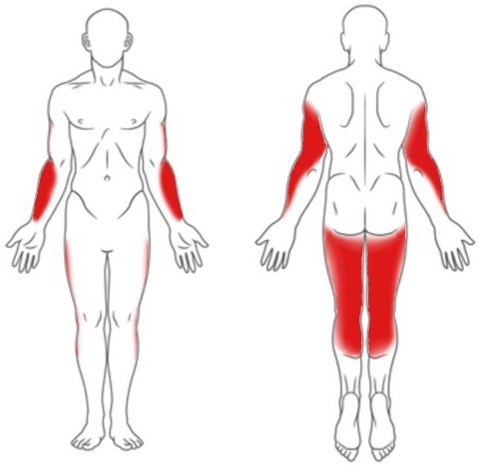
Distribution of myalgia.

**Figure 2.**
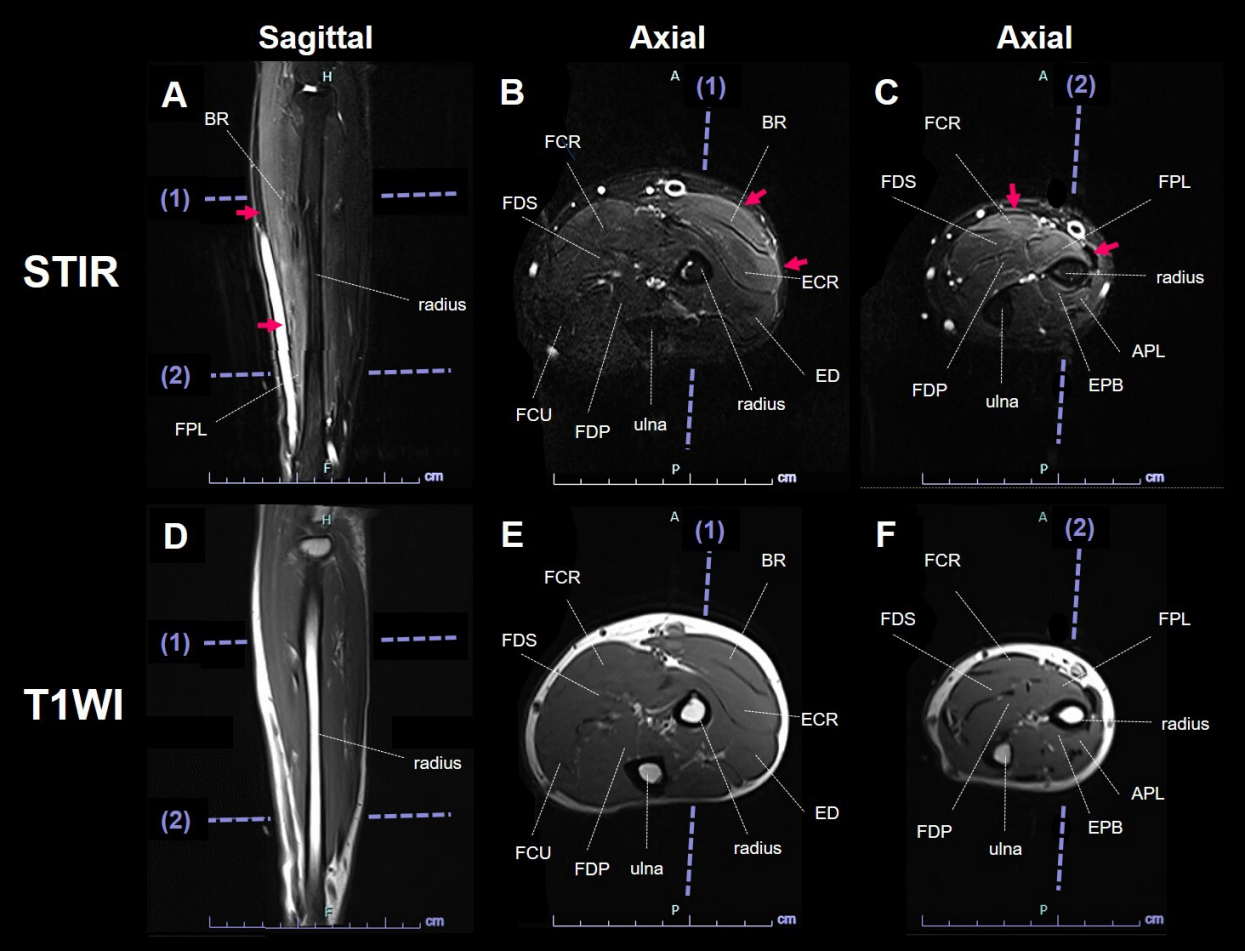
MRI of the left forearm. STIR sagittal image (A) and axial images (B, C) show hyperintensity in the muscle and fascia in the BR, ECR, FCR, and FPL muscles (A-C, arrows). The FDP muscle appeared intact. T1-weighted images show normal findings (D-F). APL: abductor pollicis longus; BR: brachioradialis; ECR: extensor carpi radialis longus and brevis; ED: extensor digitorum; EPB: extensor pollicis brevis; FCR: flexor carpi radialis; FCU: flexor carpi ulnaris; FDP: flexor digitorum profundus; FDS: flexor digitorum superficialis; FPL: flexor pollicis longus; MRI: magnetic resonance imaging; STIR: short-tau inversion recovery; TIWI: T1-weighted image.

## Article Information

### Conflicts of Interest

None

### Author Contributions

RS, NU, HT, and HM were involved in the acquisition, analysis, or interpretation of data. RS and NU drafted the manuscript. All authors contributed to the revision of the manuscript and approved the final version.

### Approval by Institutional Review Board (IRB)

Not applicable.

### Informed Consent

The patient signed informed consent forms for academic use of the data.
